# A Novel Method of Endotoxins Removal from Chitosan Hydrogel as a Potential Bioink Component Obtained by CO_2_ Saturation

**DOI:** 10.3390/ijms23105505

**Published:** 2022-05-14

**Authors:** Adrianna Banach-Kopeć, Szymon Mania, Joanna Pilch, Ewa Augustin, Iwona Gabriel, Robert Tylingo

**Affiliations:** 1 Department of Chemistry, Technology, and Biotechnology of Food, Faculty of Chemistry, Gdańsk University of Technology, 11/12 G.Narutowicza Str., 80-233 Gdansk, Poland; adrianna.banach@pg.edu.pl (A.B.-K.); robertt@pg.edu.pl (R.T.); 2 Department of Pharmaceutical Technology and Biochemistry, Faculty of Chemistry, Gdańsk University of Technology, 11/12 G.Narutowicza Str., 80-233 Gdansk, Poland; joapilch@pg.edu.pl (J.P.); ewa.augustin@pg.edu.pl (E.A.); iwona.gabriel@pg.edu.pl (I.G.)

**Keywords:** chitosan hydrogel, CO_2_ saturation, endotoxin, cytotoxicity, microbiological purity, molecular weight, viscosity

## Abstract

The article presents a new approach in the purification of chitosan (CS) hydrogel in order to remove a significant amount of endotoxins without changing its molecular weight and viscosity. Two variants of the method used to purify CS hydrogels from endotoxins were investigated using the PyroGene rFC Enzymatic Cascade assay kit. The effect of the CS purification method was assessed in terms of changes in the dynamic viscosity of its hydrogels, the molecular weight of the polymer, microbiological purity after refrigerated storage and cytotoxicity against L929 cells based on the ISO 10993-5:2009(E) standard. The proposed purification method 1 (M1) allows for the removal of significant amounts of endotoxins: 87.9–97.6% in relation to their initial concentration in the CS hydrogel without affecting the solution viscosity. Moreover, the final solutions were sterile and microbiologically stable during storage. The M1 purification method did not change the morphology of the L929 cells.

## 1. Introduction

Bioprinting is an additive manufacturing method in which thermoplastic materials and hydrogels filled with cells and distributed layer-by-layer are used to obtain a three-dimensional scaffold [1,2]. The bioinks used in bioprinting must fulfil a number of criteria, that is, biodegradable, non-cytotoxic, biocompatible, and porous, to allow the formation of new tissues and support cell growth throughout the regeneration period. In addition, cellular scaffolds must be adherent, allow for the exchange of gases and metabolites, and have adequate mechanical properties [3]. Polysaccharides of marine origin are raw materials that meet most of the 3D printing requirements and medical guidelines. Compared with polysaccharides obtained from mammals, there is a lower risk of transmitting infectious diseases. In contrast, natural polysaccharides exhibit a low immune response and good biocompatibility [4].

Chitosan (CS) is a polycationic polymer produced by the deacetylation of chitin, a part of the exoskeletons of marine invertebrates, insects, and fungal cell walls. It consists of d-glucosamine (70–90%) and *N*-acetyl-d-glucosamine (10–30%), which are linked to each other by random or block β-1,4-glycosidic bonds. The solubility of CS is determined by both the molecular weight and degree of deacetylation, which is defined as the ratio of the number of glucosamine groups to the total number of *N*-acetylglucosamine and glucosamine groups. CS is a bioactive biopolymer that is biocompatible, biodegradable, bioactive, has good mechanical strength, and can be easily modified. Additionally, the presence of amino groups in the polymer structure affects its antimicrobial properties [5]. CS has a stable and stiff crystalline structure, which makes the polymer insoluble in water, solutions at pH above 7, and in most organic solvents. In contrast, in diluted acidic solutions, the amine groups of CS become protonated and the molecules of this compound become soluble below pH 5 [6]. The problem of insufficient biocompatibility of CS, resulting from the presence of acid in its solutions, which is necessary to dissolve the polymer, has already been solved by our research team and consists of the use of saturation of microcrystalline CS sediment with the use of carbon dioxide [7]. One limitation of using CS in 3D printing is the presence of endotoxins. An additional complication is that the endotoxin content of the raw material varies, even within CS from the same manufacturer [8].

Endotoxins, also called lipopolysaccharides (LPS), are the main components of the outer cell membranes of gram-negative bacteria. In contrast to the thermolabile exotoxins produced by Vibrio cholerae, they are characterised by high thermal stability. Endotoxins, because of their excessive concentration in the bloodstream, are responsible for severe gram-negative bacterial infections, sepsis, and septic shock, which is associated with a mortality rate of 20–50%. LPS is released during cell death and cell growth and division. Despite the wide variation in endotoxin composition, owing to different bacterial stereotypes, there is one common scheme to describe them. LPS are amphiphilic molecules consisting of a hydrophilic polysaccharide moiety divided into a core and an O-specific chain, and a covalently bound hydrophobic lipid component known as lipid A. The essential structural elements that provide cytotoxic activity are 2-keto-3-deoxy-octulosonic acid (kdo), which are present in the core and lipid A [9]. Endotoxins are resistant to extreme temperature and pH conditions. Consequently, the removal of endotoxins by separation is more difficult than sterilisation. To inactivate endotoxins, it is recommended to expose them to 250 °C for a minimum of 30 min or 180 °C for at least 3 h [10]. There is a relationship between the concentration of endotoxins present in the product administered to the patient and the effects of endotoxins. Depending on the intended use of such a product, 2 limits are set: 5.0 EU/kg body weight for all preparations except those administered intraperitoneally, for which the limit is 0.2 EU/kg body weight [11]. The term EU denotes the biological activity of the endotoxins, which corresponds to 1 EU for 100 pg of standard endotoxin EC-5, 200 pg of EC-2, and 120 pg of endotoxin from *Escherichia coli* O111:B4 [10]. Owing to the unique and specific biological characteristics of endotoxins, various methods have been proposed for their removal. Currently, techniques based on the removal of endotoxins by size are used, such as gel permeation chromatography, ultrafiltration, or sucrose gradient centrifugation. Other methods are based on specific binding using ion-exchange and affinity chromatography techniques or extraction [12]. However, most of these methods have their drawbacks and limitations. Therefore, there is a need for research to develop new methods for the purification of polymers, considering their chemical structure and surface properties [13]. The limulus amoebocyte lysate (LAL) assay is currently the gold standard for endotoxin determination. This assay is used for the specific in vitro determination of endotoxins, based on a clotting cascade mediated by endotoxin activated factor C. The disadvantage of this method is that other alternative mediating pathways, such as factor G, may affect the test results only if they are activated in the presence of beta-glucans. Consequently, the use of this method for CS solutions is difficult because beta-glucan blockers present in the solution are required. Otherwise, CS will bind to the reagent, resulting in a false positive test result. In addition, the lack of defined quantities of the blocker used means that if too much is used, the compound will bind not only to the CS but also to the endotoxin, and a false positive result will be obtained [14]. In contrast to LAL, the recombinant factor C (rFC) test is more sensitive and specific for the determination of endotoxins. The reaction mechanism involves the binding of the endotoxin to rFC and its activation, which leads to the cleavage of the synthetic fluorogenic substrate, causing the solution to fluoresce [15].

The above test was used to evaluate the effectiveness of endotoxin removal from CS obtained by the carbon dioxide saturation method as a component of the cell bioink dedicated for bioprinting. The potential of the proposed purification method was also tested to ensure the microbiological purity of the CS solution, as well as its cytotoxic effect on murine fibroblasts (L929).

## 2. Results and Discussion

Considering the physicochemical and biological properties of CS, this polymer seems to be a very good candidate for producing cell bioinks for 3D printing technology. This is especially true for features such as biodegradability, biocompatibility, nontoxicity, immunogenicity, and antimicrobial activity [16]. CS in composites with collagen allows appropriate mechanical properties of cell scaffolds to be obtained while maintaining cell adhesion and proliferation [17]. Controlled dosing of bioink in a 3D printer requires liquid CS. Unfortunately, to dissolve the polymer, it is necessary to use an acid that significantly reduces the biocompatibility of the CS hydrogels. Such a limitation for this form of CS can be overcome by saturating the suspension of microcrystalline CS sediment with carbon dioxide, which increases the pH of the obtained hydrogels from 3–4 to 6.5, thus increasing their biocompatibility [7]. This method uses sodium hydroxide precipitation and autoclaving to obtain a sterile CS-CO_2_. Alkaline environments and high temperatures are also factors used in the removal of bacterial toxins from raw materials [18]. Therefore, in this work, we present two variants of a common method of obtaining CS with increased biocompatibility and, at the same time, devoid of bacterial toxins (2.1). We assessed their effectiveness in terms of efficiency of toxin purification, changes in molecular weight, microbiological purity, and cytotoxicity against L929 mouse fibroblasts.

### 2.1. Effect of the Method of Chitosan Hydrogel Purification on LPS Content

**Figure 1:**
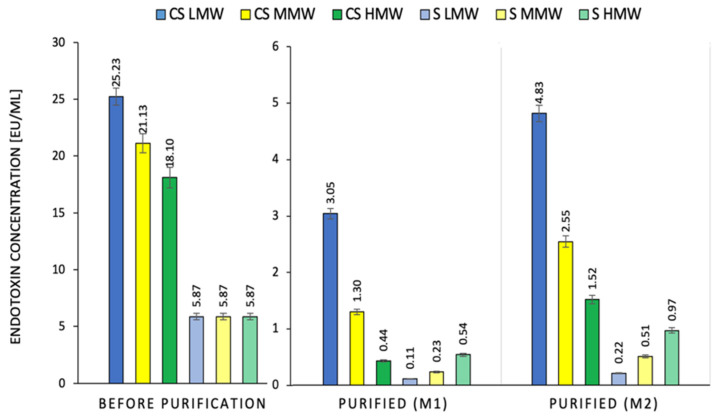
Comparison of endotoxin concentration in CS hydrogels: low molecular weight (LMW), medium molecular weight (MMW), and high molecular weight (HMW) and standard endotoxin solutions (S) after contact with CS of different molecular weights, before and after purification using M1 or M2 method (*n* = 3, *p* < 0.05).

**Figure 2:**
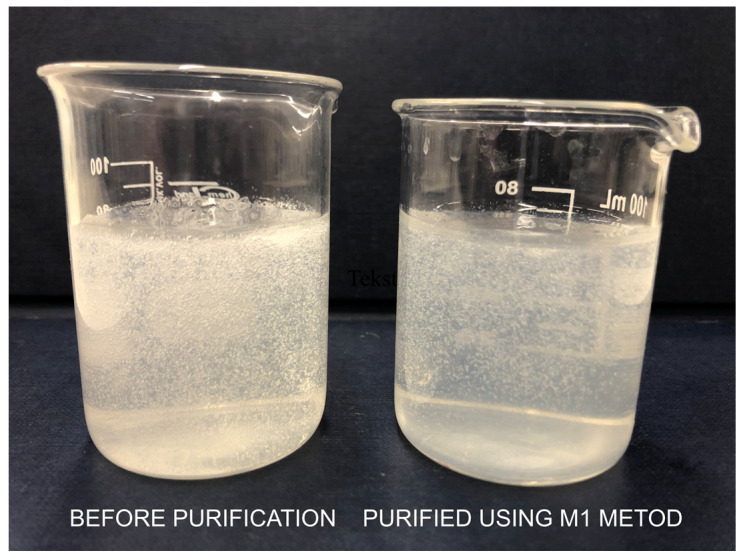
Comparison of the appearance of chitosan hydrogel samples before and after treatment with M1 method.

The content of bacterial endotoxins in the LMW, MMW and HMW CS hydrogels ranged from 18.1–25.2 EU/mL (Figure 1). The use of the M1 method for their purification reduced the endotoxin content by 87.9%, 93.8%, and 97.6%, respectively, in relation to their initial concentration in the CS hydrogel. Purification by M2 lowered the endotoxin levels in the hydrogels of the LMW, MMW, and HMW samples by 80.9%, 87.9%, and 91.6%, respectively. This indicates a higher toxin removal efficiency with the M1 method (6–7%). The visual effect of treatment with the M1 method is shown in Figure 2. It confirms that the purification by this method does not affect the appearance of the chitosan hydrogel. It may also appear that the purification efficiency increases with increasing molecular weight. This dependence was observed for both the purification methods. The higher efficiency of LPS extraction in the M1 method may have resulted from the increased contact of the polymer with chloroform. This solvent was used here to wash the hydrogel and not the CS sediment, as is the case for M2 (Figure 1). In the scientific literature, CS, due to its polycationic nature, is instead perceived as a compound that catches bacterial toxins with a negative charge [19]. Therefore, the phenomenon of a higher affinity of endotoxin for low molecular weight CS was confirmed in the test with its standard (S) (Figure 1). The addition of the same mass sample of LMW, MMW, and HMW CS to the LPS standard solution at a 5.87% concentration resulted in the binding of the toxin with a similar effect. This means that the reduction in the LPS concentration of the standard solution was 90.7%, 96.0%, and 98.1% for M1 and 83.6%, 91.3%, and 96.3% for M2, respectively.

In comparison, Lebre et al. proposed a method for purification of CS particles based on dissolution and precipitation of the solution under strongly acidic and basic conditions. Based on the gel-clot LAL assay with a detection limit of 0.125 EU/mL, it was shown that the method can be used for endotoxin removal; however, due to its limitations, follow-up tests are needed to confirm the test result [18].

Similar results were obtained by Davydova et al., who confirmed that CS with a molecular mass of 20 kDa had a higher affinity for LPS than chitosan with a molecular mass of 140 kDa. However, precise determination of the molecular mechanism of the interaction between CS and endotoxins is difficult because of the variability of both raw materials. Endotoxins are heterogeneous and capable of forming polydisperse aggregates of high molecular weight in aqueous solutions, and their organization depends on the length of O-specific lipopolysaccharide chains [20].

### 2.2. Effect of the Method of Chitosan Hydrogel Purification on the Molecular Weight of Polymer and Hydrogel Viscosity

**Figure 3:**
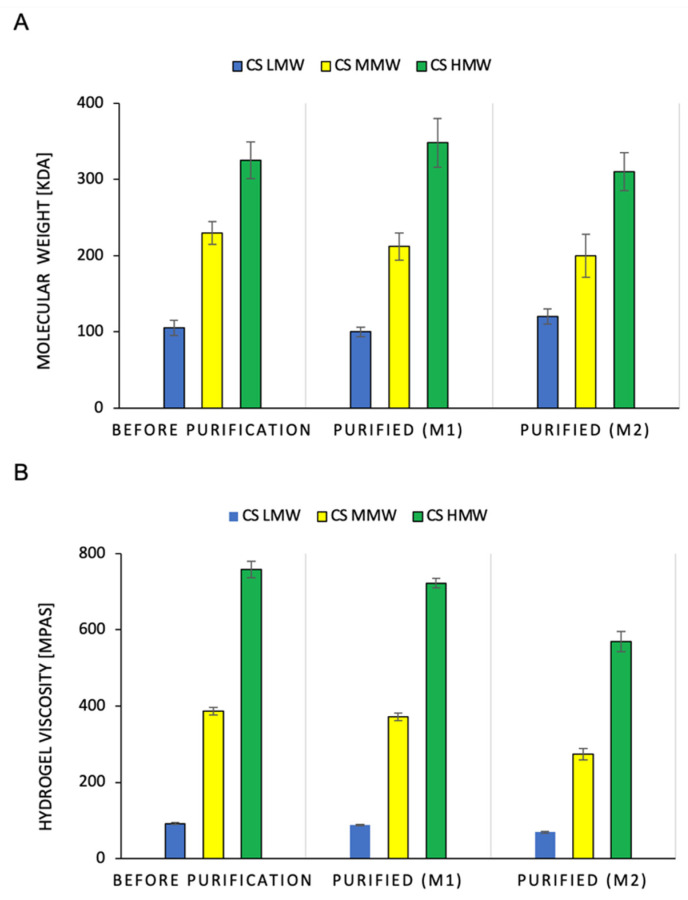
Comparison of molecular weight of chitosan polymer (**A**) and chitosan hydrogel. viscosity (**B**) with LMW, MMW and HMW, before and after purification using M1 or M2 method (*n* = 3, *p* < 0.05).

Figure 3 shows the effect of the purification methods on the change in the molecular weight of CS and the viscosity of the hydrogels. Regardless of the purification method used, the molecular mass of the CS did not significantly change. This applies to the low, medium, and high molecular weights of the polymers (Figure 3A). It was observed that the M1 purification method reduced the viscosity of the CS hydrogel by 3.6–4.7%. In contrast, for the M2 method, the losses were much greater, ranging from 24.8% to 29% (Figure 3B). The obtained results indicate that the CS chain did not degrade during purification, which could be initiated by the action of high temperature (Figure 6). A significant reduction in the viscosity of the hydrogels after M2 purification was due to the loss of CS during the washing of the sediment with chloroform. Chloroform physically delaminated the CS precipitate obtained by precipitation with NaOH. In the M1 method, this was not the case because the extraction of the toxins with chloroform occurred immediately after the dissolution of CS in the hydroxyacetic acid solution. Under these conditions, chloroform spontaneously separates from the aqueous layer containing the polymer.

Martini et al. measured the degradation rate of different CS hydrogels in dynamic sweep test mode by monitoring several parameters, such as temperature, CS concentration, dynamic viscosity, and polymer molecular weight, and concluded a clear dependence between these parameters. The results demonstrate that there is a direct relationship between solvating conditions and CS backbone integrity overtime in an acidic environment [21]. On the other hand, in the proposed CS purification procedure, the temperature treatment (12 °C, 15 min) was subjected to the CS sediment suspended in an appropriate amount of distilled water and no degradation of the polymer chain was observed. It is highly probable that the rate of CS degradation is mainly determined by the presence of acid in the environment and only by other factors such as concentration and molecular weight. The presence of acid in CS materials mainly causes the hydrolysis of the polymer to lower molecular weight without significantly affecting the degree of deacetylation and polydispersity [22]. We observed a similar phenomenon during the attempt to thermally degrade dry CS-poly(vinyl acid) composites, in which the elimination of the acid from the CS material significantly increases its stability during thermal treatment, even when it is used as a filler for thermoplastic materials [1].

### 2.3. Effect of the Method of Chitosan Hydrogel Preparation on Microbiological Purity

TVC is a microbiological parameter commonly used to assess the total number of bacteria, yeasts, and molds. The sterilization step applied by autoclaving the chitosan solutions in the M1 and M2 methods yielded sterile solutions, for which no colony growth was observed (Table 1).

In addition, the solutions showed microbiological stability when stored at 4 °C for 1 month. On the other hand, for the control sample, which was an unsterilised and unpurified chitosan solution, an increase in TVC was observed, which was 1.6 × 10^3^ CFU/g (colony-forming unit per gram) was observed on the first day. After 2 weeks and 1 month of storage at 4 °C, the number increased to 2.5 × 10^3^ and 3.3 × 10^4^ CFU/g, respectively. The results showed that the chitosan hydrogels obtained by the M1 and M2 methods could be successfully stored for further distribution without the need for reserialization.

The applied storage conditions of chitosan hydrogels, such as 4 °C temperature and previous sterilization, are a consequence of problems regarding the stability of chitosan products which limits their further application. The rate of hydrolysis of chitosan chains is consistent with first-order kinetics; therefore, storage at low temperatures of 2–8 °C is recommended [23]. In addition, despite the broad-spectrum antimicrobial activity of chitosan, its activity is dependent on its physicochemical properties, such as pH, degree of deacetylation, molecular weight, derivatives, and the type of microorganism [24]. Therefore, there is a need for sterilization to ensure the safety of chitosan hydrogels. Based on the study by San Juan et al., it was found that autoclaving physical chitosan hydrogels did not significantly change the macromolecular structure of the polymer. In addition, as it is one of the most convenient sterilisation procedures, it can be used to sterilise physical chitosan hydrogels after preparation [25]. Sterilisation by autoclaving can be successfully applied to the M2 method of chitosan purification because it does not significantly affect the depolymerisation of chitosan, as evidenced by small changes in the viscosity of the solutions during purification.

### 2.4. Effect of the Method of Chitosan Hydrogel Preparation on Cytotoxicity

**Figure 4:**
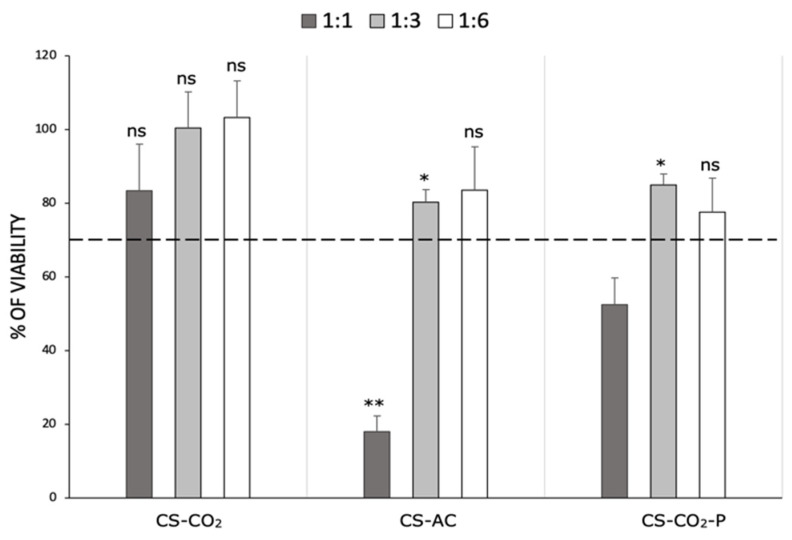
The cytotoxicity of chitosan hydrogels: chitosan dissolved by CO_2_ saturation method (CS-CO_2_), chitosan dissolved in hydroxyacetic acid (CS-AC), and chitosan hydrogel purified by M1 method (CS-CO_2_-P) following 24 h of incubation at different dilution factors (1:1, 1:3, and 1:6) against L929 cells. Data are expressed as the mean ± standard deviation of three independent experiments. The results were analyzed by two-way ANOVA with Dunnett’s multiple comparisons vs. Control: ns (not statistically significant, *p* > 0.05), * *p* < 0.01, ** *p* < 0.001.

The MTT assay was used to investigate the cytotoxicity of the chitosan hydrogels against adult mouse fibroblast L929 cells at different dilutions (1:1, 1:3, and 1:6). The obtained results were expressed as the percentage of viable cells compared to the control without the materials. The cytotoxic effect was determined for MMW CS-AC, CS-CO_2_ [7], and chitosan hydrogel purified by M1 method, defined as the optimal purification procedure based on previous results (CS-CO_2_-P). According to Huang et al., molecular weight does not affect the cytotoxicity of chitosan; therefore, only a polymer with an average molecular weight was used for the determination [26]. The data presented in Figure 4 clearly shows that the CS-CO_2_ hydrogel was not toxic to L929 cells, regardless of the dilution used. According to ISO 10993-5:2009(E), this material exhibits good biocompatibility. Significant cell growth inhibition of the CS-AC sample was observed only at a dilution 1:1 (80% compared to the control). In turn, CS-CO_2_-P at the same dilution inhibited L929 cell growth by approximately 50% compared to cells without the hydrogel. The remaining diluent fractions (1:3 and 1:6) of the CS-AC and CS-CO_2_-P samples were non-toxic (>70% viability) to L929 cells. However, these materials inhibited cell growth more efficiently than CS-CO_2_ did at the same diluent fraction. This proves that chitosan dissolved in carbonic acid demonstrated good biocompatibility.

**Figure 5:**
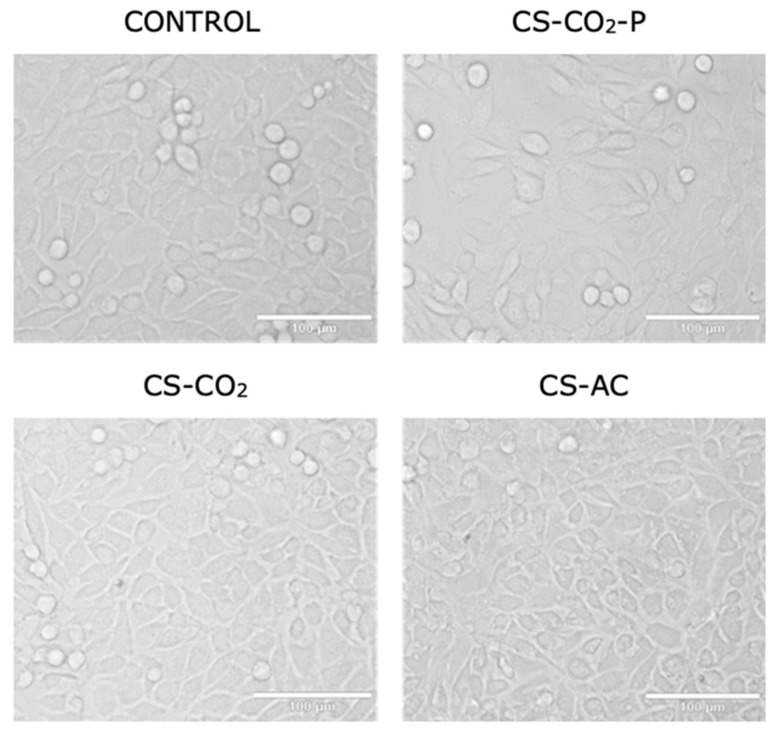
Representative image at 200× magnification of L929 cells following 24 h of treatment with chitosan hydrogels: CS-CO_2_, CS-AC, and CS-CO_2_-*p* at 1:3 diluent factor. The scale bar is 100 µm.

The cytotoxicity data were confirmed by morphological examination of L929 cells treated with the studied chitosan derivatives diluted at 1:3 for 24 h. This dilution was chosen because the viability of the cells reached 70% following treatment with chitosan derivatives compared to that of the control. Figure 5 shows that L929 cells presented unchanged morphology (maintaining their fibroblast shape) following treatment with CS-CO_2_-P and CS-CO_2_ chitosan hydrogels compared to the control. In contrast, treatment with the CS-AS hydrogel resulted in morphological changes (the cells were shrunken and did not retain their fibroblast morphology). Endotoxins have a variety of effects on cell cultures that can result in pyrogenic responses ranging from fever and chills to irreversible and fatal septic shock, and may result from endotoxin levels in final products and the influence of endotoxins on the expression system. Nalbantsoy et al. showed that endotoxins do not equally affect cell cultures. Some cell cultures lack the appropriate endotoxin receptors and may only be sensitive to very high levels of LPS. Therefore, such a risk must be eliminated in the case of chitosan hydrogels, which must meet the requirements for bioinks in the production of cell scaffolds using the bioprinting method [27].

## 3. Materials and Methods

### 3.1. Materials

Low molecular weight CS (LMW) with 50–190 kDa molecular weight and 75–85% deacetylation degree, medium molecular weight CS (MMW) with 190–310 kDa molecular weight and 75–85% deacetylation degree, and high molecular weight CS (HMW) with 310–390 kDa molecular weight and above 75% deacetylation degree were purchased from Merck (Darmstadt, Germany). 3-(4,5-dimethylthiazol-2-yl)-2,5-diphenyltetrazolium bromide (MTT), streptomycin, and penicillin were purchased from Sigma-Aldrich, St. Louis, MO, USA. IPA was purchased from POCH (Gliwice, Poland). MilliQ water was used to prepare all the aqueous solutions (Milli-Q^®^ IQ 7005 Water Purification System, Millipore, Boston, MA, USA). All other reagents were of analytical grade or higher. Chloroform, hydroxyacetic acid, and sodium hydroxide were purchased from Avantor Performance Materials Poland S. A. (Gliwice, Poland). The carbon dioxide used to saturate the CS precipitate was obtained from Linde Gaz Polska Sp. z o. o. (Gdańsk, Poland). Tryptic soy agar (TSA) medium was purchased from Merck (Darmstadt, Germany).

### 3.2. Chitosan Hydrogels Preparation and Purification

**Figure 6:**
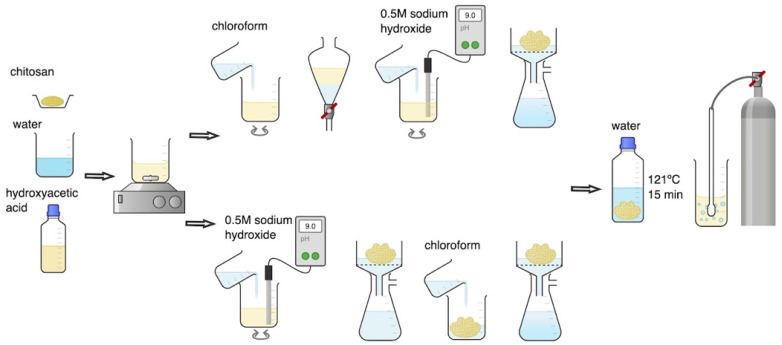
Scheme of chitosan (CS) hydrogel purification using M1 (top) and M2 (bottom) methods.

CS hydrogels were prepared with three molecular weight variants: low (LMW), medium (MMW), and high (HMW). The concentration of endotoxins before purification was determined in 1% (*m*/*v*) CS hydrogels obtained by direct dissolution of the polymer in 0.1 M hydroxyacetic acid solution during mechanical stirring at 300 RPM (RA 2020, Heidolph Instruments GmbH & Co. KG, Kelheim, Germany). The effectiveness of endotoxin removal from hydrogels was assessed after their purification using the M1 and M2 methods presented in Figure 6. Both purification methods constitute a modification of the CS dissolution method by saturating the water suspension of the microcrystalline sediment with carbon dioxide [7].

In the M1 purification method, 100 mL of CS dissolved in hydroxyacetic acid was transferred to a 250 mL separating funnel and washed 3 times with 50 mL of chloroform. After separation of chloroform, the CS hydrogel was transferred to a beaker, a 0.5 M sodium hydroxide solution was added during mixing until a pH value in the range of 9–10 was reached. This was equivalent to the complete precipitation of CS in the microcrystalline form. The precipitated CS was filtered using a seepage kit under reduced pressure and washed several times with distilled water until the pH of the rinsing water reached a value equal to 7.0. Finally, the precipitated CS was weighed, sterilised at 121 °C for 15 min (Systec VB-50, De Ville, Raszyn, Poland), and suspended in an amount of free endotoxin distilled water to obtain a 1% (*m*/*v*) CS solution. The CS suspension was homogenized at 10,000 rpm for 3 min (Silent Crusher M, Heidolph Instruments GmbH & Co. KG, Kelheim, Germany) and then saturated with CO_2_ by simultaneous mechanical mixing using a hollow shaft stirrer for gas saturation (BIO-MIXBMX-10, Gdańsk, Poland) until completely dissolved.

In the M2 purification method, 100 mL of CS dissolved in hydroxyacetic acid was first treated with 0.5 M sodium hydroxide solution during mixing until a pH value in the range of 9–10 was reached. The precipitated CS was then filtered using a seepage kit under reduced pressure, washed 3 times with 50 mL of chloroform, and several times with distilled water until the pH of the rinsing water reached a value equal to 7.0. Finally, the precipitated CS was weighed, sterilised at 121 °C for 15 min (Systec VB-50, De Ville, Raszyn, Poland), and suspended in an amount of free endotoxin distilled water to obtain a 1% (*m*/*v*) CS solution. The CS suspension was homogenized at 10,000 rpm for 3 min (Silent Crusher M, Heidolph Instruments GmbH & Co. KG, Kelheim, Germany) and then saturated with CO2 by simultaneous mechanical mixing using a hollow shaft stirrer for gas saturation (BIOMIXBMX-10, Gdańsk, Poland) until completely dissolved.

### 3.3. Determination of Endotoxins Concentration

The endotoxin concentration in CS hydrogels was determined using the PyroGene rFC Enzymatic Cascade assay (Lonza Group Ltd., Visp, Switzerland). The solutions for testing endotoxin concentrations were applied to a 96-well plate, which was incubated in a microplate reader at 37 °C. The fluorescence of the solution was measured at an excitation/emission wavelength of 380/440 nm (Spark^®^ multimode, microplate reader, TEKAN, Maennedorf, Switzerland). The endotoxin concentration in solutions was determined based on a previously determined sight curve in the range of concentration 0.5–5.0 EU/mL of *E. coli* 055: B5, E50-643 endotoxin. This assay was used to perform two tests. First, 1% CS solutions in hydroxyacetic acid (before purification) and 1% CS solutions purified using the M1 and M2 methods were tested. The second part of the test was used to evaluate the endotoxin binding capacity of unpurified and purified 1% CS solution using the M1 and M2 methods. For this purpose, a standard solution of endotoxin *E. coli* 055: B5, E50-643 with a concentration of 5.87 EU/mL was prepared. Next, 100 mg of the tested CS was added to o 2 mL of the standard solution and shaken for 15 min at 25 °C (Unimax 1010 with Incubator 1000, Heidolph Instruments GmbH & Co. KG, Kelheim, Germany). The assay solution was taken from the insoluble precipitate of the polymer after centrifuging the samples at 3000× *g* for 5 min. (MPW-260; Labfunk, Kluczbork, Poland). Dry CS preparations after purification used for this determination were obtained by freeze-drying: 0.94 mbar, condenser temperature: −78 °C (Christ Alpha 2-4 LD Plus, Donserv, Warsaw, Poland).

### 3.4. Determination of Chitosan Molecular Weight and Hydrogel Viscosity

To determine the dynamic viscosity of 1%, CS solutions were introduced into a measuring cell equipped with a temperature-controlled bath (Brookfield Digital Model DVIII Ultra viscometer (Middleborough, MA, USA). The measurements were carried out using SC4-27 and SC4-25 spindles at a temperature of 25 °C and shear stress of 22.00 1/s. All rheological measurements were performed in triplicate. The viscosity average molecular weight was identified by viscosimetric measurements using a Ubbelohde capillary viscometer type 531/10 according to the method proposed by Yacob et al. [28]. This value was calculated from [η] = KMα equation, where K = 9.66 × 10^−5^ (dm^3^/g) and a = 0.742 determined in 0.15 M ammonium acetate and 0.2 M acetic acid solutions at 25 °C.

### 3.5. Purity and Microbiological Stability

A control sample of 1% CS (MMW) in hydroxyacetic acid and 1% CS (MMW) purified using the M1 and M2 methods was stored at 4 °C for 1 month. Total viable count (TVC) was determined immediately after the preparation of the solutions and after 14 and 30 days of storage. To determine the TVC, serial dilutions were made and samples from each tube were plated on a solid TSA medium using the pour plate method. Plates were incubated at 37 °C for 48 h. After incubation, bacterial colonies grown on Petri dishes were counted.

### 3.6. Determination of Cytotoxicity

#### 3.6.1. Cell Culture

The adult mouse fibroblast L929 cell line was purchased from the American Type Culture Collection (ATCC; Manassas, VA, USA) and tested for mycoplasma using a Universal Mycoplasma Detection Kit, ATCC-30-1012 K (ATCC). The L929 cell line was cultured in low-glucose Dulbecco’s modified Eagle’s medium (DMEM; Sigma-Aldrich, St. Louis, MO, USA) supplemented with 10% foetal bovine serum (FBS; Biowest, Riverside, MO, USA), 100 µg × mL^−1^ streptomycin, and 100 units × mL^−1^ penicillin. Cells were incubated in a humidified atmosphere containing 5% CO_2_ at 37 °C. All of the experiments were performed with cells in the exponential growth phase.

#### 3.6.2. Cell Viability and Morphology Assessment

The in vitro cytotoxicity of the foams was assessed using the 3-(4,5-dimethylthiazol-2-yl)-2,5-diphenyltetrazolium bromide (MTT) assay, according to ISO 10993-5:2009(E). Briefly, L929 cells (1 × 10^4^ cells/well) were seeded in 96-well plates, and 100 μL of culture medium only (blank) was dispensed into the peripheral wells. After 24 h of preincubation at 37 °C in a humidified 5% CO_2_ atmosphere, the culture medium was removed and replaced with a fresh medium containing different concentrations of sample, positive control, or blank. Following 24 h of incubation, the culture medium was removed, and 50 μL of the MTT solution (1 mg × mL^−1^ in medium without supplements and phenol red) was added to each well and incubated for the next 2 h at 37 °C in a humidified 5% CO_2_ atmosphere. Furthermore, media containing the MTT solution were removed, and the formazan crystals were dissolved in 100 µL of isopropanol and shaken for 10 min. Absorbance at 540 nm was measured using a microplate reader (iMark™, Bio-Rad, Warsaw, Poland). The results were repeated at least three times. was obtained from three independent experiments (*n* = 3).

The morphology of L929 cells following treatment with the CS compounds was evaluated using a light microscope. Briefly, L929 cells were seeded at a 60 mm plate with coverslips at a density of 1 × 10^6^ and incubated overnight. Then, the culture medium was removed and replaced with fresh medium containing CS compounds such as: chitosan hydrogel in CO_2_ (CS-CO_2)_, chitosan dissolved in hydroxyacetic acid (CS-AC) and purified chitosan hydrogel (CS-CO_2_-P) at a 1:3 dilution factor for 24 h. Finally, the cells were observed using a light microscope (Olympus IX83, Tokyo, Japan; objective 200× magnification).

### 3.7. Statistical Analysis of the Data

STATISTICA software (StatSoft, Inc., Tulsa, OK, USA) was used for all of the analyses. Statistical significance was set at *p* < 0.05. All of the data reported are based on the means of 3 replicates (*n* = 3) or 5 replicates (*n* = 5) in the case of mechanical tests. Experimental results are expressed as mean ± standard deviation (SD). Student’s t-test and one-way analysis of variance (ANOVA) were applied. Differences were considered statistically significant at *p* < 0.05.

## 4. Conclusions

In line with this hypothesis, we assumed that the procedure of obtaining a chitosan hydrogel by saturating the aqueous suspension of its microcrystalline sediment, combined with the stages of washing out toxins with chloroform, will allow for the preparation of biocompatible and endotoxin-free solutions, which can be used as the main component in the preparation of bioinks for creating personalised cell scaffolds using the bioprinting method. The obtained results confirmed that 1 of the proposed methods (M1) is characterised by a high purification efficiency of the chitosan hydrogel (88–98%) without affecting the solution viscosity after purification, as well as the molecular weight of the polymer. The method also allowed us to obtain a microbiologically pure hydrogel immediately after treatment and after 30 days of refrigerated storage. The results of cytotoxicity tests were also promising. They clearly showed that the method of CO_2_ saturation (CS-CO_2_) allows for the production of a non-cytotoxic chitosan hydrogel against mouse fibroblast cells. The addition of chloroform slightly reduced the viability of the cells, which was evident in the samples with the lowest dilution. However, the lack of changes in cell morphology and the higher survival of the L929 line in the CS-CO_2_-P sample compared to the CS-AS sample indicate that the method seems to be promising. Increasing the viability of cells in contact with the CS-CO_2_-P sample will probably be achieved by extending the duration of carbon dioxide sowing. The results obtained will be the basis for further tests on the regeneration of skin tissue with the use of chitosan.

## Figures and Tables

**Table 1:** Effect of Purification Method on Total Viable Count during the 30-Days Storage Period at 4 °C (*n* = 3).

Storage Period (d)	Total Viable Count (CFU/g)		
	Control	M1	M2
0	1.6 × 10^3^	ND	ND
15	2.5 × 10^3^	ND	ND
30	3.3 × 10^4^	ND	ND

ND = not detected.

## Data Availability

Not applicable.
